# Engaging Villages as Key Partners for Healthy Aging Outcomes Research: A Framework for Capacity Development

**DOI:** 10.1093/geroni/igaf122.712

**Published:** 2025-12-31

**Authors:** Natalie Pope, Emily Greenfield

**Affiliations:** Rutgers University, New Brunswick, New Jersey, United States; Rutgers University, New Brunswick, New Jersey, United States

## Abstract

There is sustained enthusiasm for Villages as a community-centered model to promote the health and well-being of people as they age in their communities. Yet there are many challenges to advancing rigorous empirical evidence on the outcomes of Villages, including their small size, grassroots nature, variable implementation, and multiple components. As part of a larger engagement project, we aimed to develop a framework for capacity development toward outcomes research with Villages. We used qualitative data from virtual summits with approximately 400 Village participants across the United States and several other countries, including Village members, volunteers, staff, and partners. Data were primarily from discussion groups that asked participants to reflect on how Villages influence healthy aging, how Villages connect with broader health systems, and conditions for high-quality research partnerships. Through iteratively coding the data, our analysis identified four thematic categories for capacity development among Villages to engage in healthy aging outcomes research: (a) understanding and motivation for research (e.g., the desire to engage in research that “proves” the benefits of Villages), (b) cultivating partnerships (e.g., with other Villages and researchers), (c) theorizing Village interventions (including Villages as an intervention, as well as interventions embedded within Villages), and (d) procuring data systems and research protocols. Findings indicate how capacity development is not only a matter of technical skills and resources, but also encompasses social capital, human motivation, and theory development. The framework can assess readiness and capacity needs for Villages and similar community-based organizations to engage in healthy aging outcomes research.

